# Serum COPA for the diagnosis and immunotherapeutic monitoring of hepatocellular carcinoma: a retrospective study

**DOI:** 10.3389/fphar.2026.1908605

**Published:** 2026-08-21

**Authors:** Ranliang Cui, Yue Cao, Li Ren, Yueguo Li

**Affiliations:** 1 Department of Laboratory, Tianjin Medical University Cancer Institute and Hospital, National Clinical Research Center for Cancer, Tianjin Key Laboratory of Digestive Cancer, State Key Laboratory of Druggability Evaluation and Systematic Translational Medicine, Tianjin’s Clinical Research Center for Cancer, Tianjin, China; 2 Department of Laboratory, Tianjin First Central Hospital, School of Medicine, Nankai University, Tianjin, China

**Keywords:** biomarker, COPA, diagnosis, hepatocellular carcinoma, immunotherapy

## Abstract

**Background and Purpose:**

Investigating the assessment of innovative serum indicators with elevated sensitivity and specificity is crucial for enhancing the early diagnosis and prognosis of HCC (Hepatocellular carcinoma). This study aimed to evaluate the efficacy of COPA (Coatomer Subunit Alpha) in diagnosing hepatocellular carcinoma and assessing the effectiveness of immunotherapy.

**Methods:**

This study included two cohorts: a diagnostic cohort comprising 81 healthy controls, 118 patients with benign liver diseases, and 81 patients with pathologically confirmed HCC, and an immunotherapy cohort comprising 131 HCC patients treated with PD-1/PD-L1 inhibitors. Serum COPA was measured by ELISA, while AFP and PIVKA-II were measured by electrochemiluminescence. Diagnostic performance and immunotherapy monitoring efficacy were then evaluated.

**Results:**

COPA is upregulated in serum and HCC tumor samples. The ROC analysis indicated that the diagnostic efficacy of COPA (AUC = 0.911) surpassed that of traditional biomarkers. Importantly, COPA retained favorable diagnostic performance in both AFP-negative and PIVKA-II-negative HCC patients. Dynamic monitoring revealed that when comparing PIVKAII and AFP with imaging-based efficacy assessments, serum COPA changes demonstrated superior consistency pre- and post-immunotherapy (Kappa of COPA = 0.73, *P* < 0.01). COPA expression was significantly elevated in HCC tissues compared with adjacent non-tumor tissues.

**Conclusion:**

This study established that COPA outperforms AFP and PIVKAII in diagnostic and immunotherapeutic monitoring reliability, offering a novel technique for the precise diagnosis and treatment of hepatocellular carcinoma.

## Introduction

Hepatocellular carcinoma (HCC) is a major cause of cancer-related mortality worldwide and is characterized by complex pathogenesis and poor prognosis. In China, HCC remains one of the leading malignant tumors, with a 5-year survival rate of less than 20% (Wang et al., 2024; Han et al., 2024). Current diagnosis mainly relies on imaging modalities (ultrasound, CT/MRI) and serum biomarkers, including alpha-fetoprotein (AFP) and abnormal prothrombin (PIVKA-II). However, AFP shows limited sensitivity (40%–60%) for early-stage HCC and may produce false-positive results in patients with chronic liver disease, while imaging methods remain challenging for detecting early microscopic lesions and heterogeneous tumors (Zeeuw et al., 2025; Tayob et al., 2023; Singh et al., 2023).

Immune checkpoint inhibitors (ICIs), particularly PD-1/PD-L1 inhibitors, have improved survival outcomes in patients with unresectable HCC. The phase III trial of atezolizumab plus bevacizumab demonstrated significant overall survival benefits; however, heterogeneous responses and treatment resistance remain major challenges in clinical practice (Finn et al., 2020; Wang et al., 2023; Ji et al., 2023). Therefore, identifying immune-related biomarkers for predicting and monitoring therapeutic responses has become an important research focus.

Coatomer subunit alpha (COPA), located on chromosome 1q23.2, encodes the α-COP protein, a key component of the coatomer complex involved in ER-to-Golgi vesicular transport and protein sorting. Beyond its role in intracellular trafficking, COPA has been implicated in tumorigenesis through regulation of RNA editing and immune-related pathways. Aberrant COPA-dependent A-to-I RNA editing has been reported in multiple cancers and is associated with poor prognosis (Song et al., 2021; Takeda et al., 2019; Thomas, 2016). Moreover, COPA mutations can activate the STING signaling pathway, affecting type I interferon production and immune microenvironment homeostasis (Watkin et al., 2015; Steiner et al., 2022; Lepelley et al., 2020).

In HCC, multi-omics studies have demonstrated that COPA expression is associated with tumor progression and prognosis (Song et al., 2025). COPA may influence immunotherapy responses by regulating metabolic pathways, antigen-presenting cell functions, and immune-related processes (Hou et al., 2023; Bao et al., 2022). Furthermore, COPA alterations, including I164V mutation-induced PI3K/AKT/mTOR pathway activation and abnormal RNA editing, have been implicated in HCC progression and ICI response prediction (Yablonovitch et al., 2017; Wa et al., 2023). These findings suggest that COPA may represent a potential molecular link between HCC development and immunotherapy resistance (Song et al., 2021; Song et al., 2025).

This study examined COPA’s therapeutic significance within the “genome-proteome-functional network” framework by merging ELISA data with bio-confidence analysis. The objective is to deliver a straightforward, user-friendly, prompt, and precise marker for diagnosing HCC and assessing treatment effectiveness.

## Materials and methods

### Patients and specimens

**FIGURE 1:**
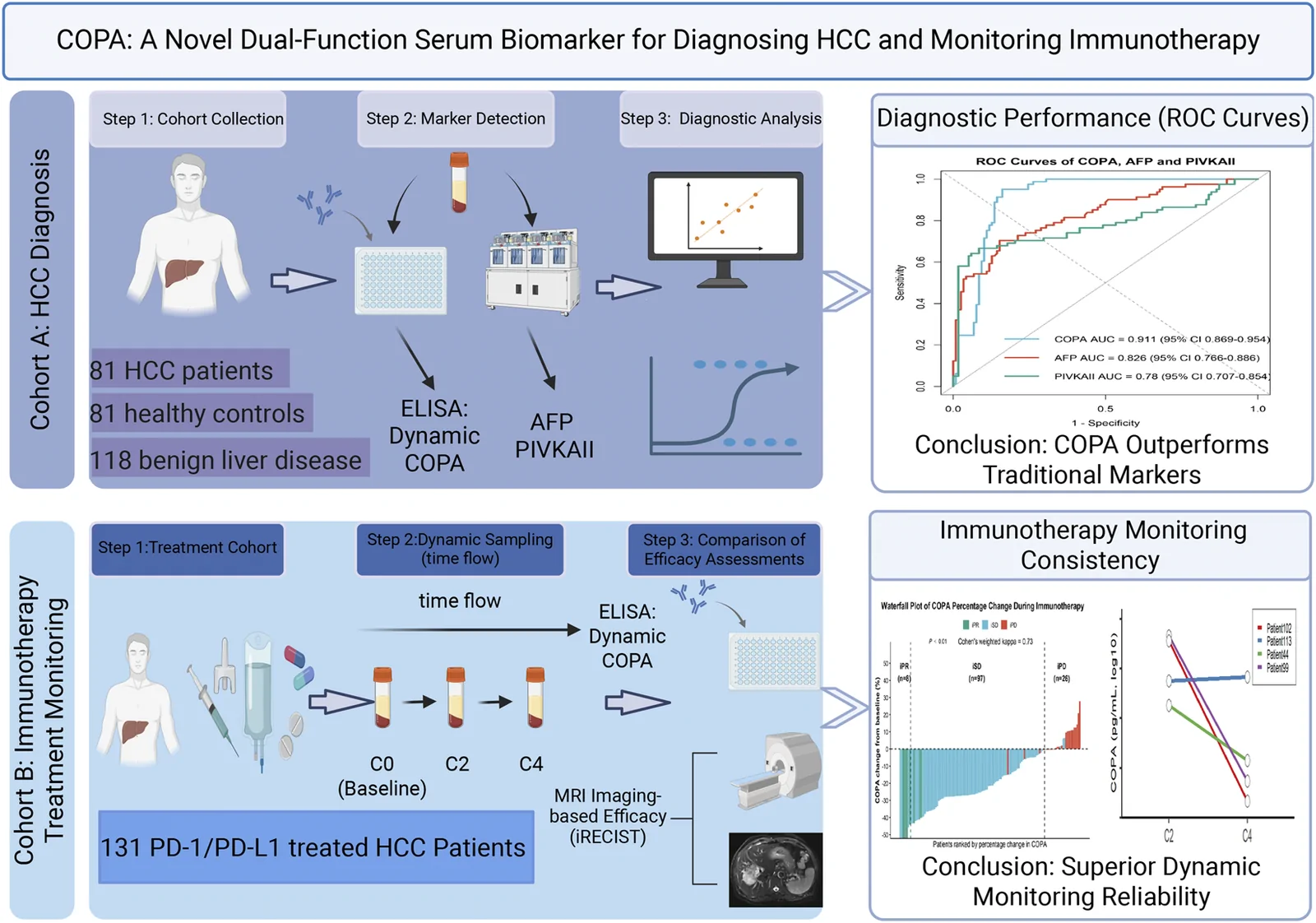
Graphical Abstract. Overview of the study design and main findings. Upper panel: Diagnostic cohort comprising 81 patients with HCC, 118 patients with benign liver disease, and 81 healthy controls. Serum COPA levels were measured by ELISA, while AFP and PIVKA-II were determined using routine clinical assays. ROC analysis demonstrated that COPA achieved superior diagnostic performance (AUC = 0.911) compared with AFP and PIVKA-II for distinguishing HCC from control subjects. Lower panel: Immunotherapy cohort comprising 131 patients with HCC receiving first-line PD-1/PD-L1 inhibitor-based systemic therapy. Serial blood samples were collected at baseline (C0), after two treatment cycles (C2), and after four treatment cycles (C4). Dynamic changes in serum COPA levels were closely associated with treatment responses assessed according to the iRECIST criteria, supporting the potential of COPA as a longitudinal biomarker for immunotherapy monitoring.

Between September 2024 and January 2025, we retrospectively collected clinical data and peripheral blood samples from two independent cohorts, including a diagnostic cohort and an immunotherapy cohort, at Tianjin Medical University Cancer Hospital. Using the electronic medical record system, HCC patients were identified and screened separately according to predefined inclusion and exclusion criteria for each cohort. Following the screening process, 81 patients were included in the diagnostic cohort, and 131 patients were included in the immunotherapy cohort. The overall study design and workflow are summarized in the Graphical Abstract (Figure 1). The detailed patient selection process is illustrated in the study flow diagram (Supplementary Figure S1).

For the diagnostic cohort, we included 81 patients with pathologically confirmed HCC, 118 patients with chronic liver disease (CLD; including 44 patients with liver fibrosis and 74 patients with liver cirrhosis), and 81 healthy controls. Healthy controls and patients with CLD were combined as the control group for diagnostic analyses. Patients were eligible if they had a pathologically confirmed diagnosis of HCC, had not received any previous anti-tumor treatment before blood sample collection, and had complete clinical and laboratory data. Patients with other malignancies, severe systemic diseases, active infections, or incomplete clinical information were excluded.

For the immunotherapy cohort, we included 131 patients with pathologically confirmed HCC who received PD-1/PD-L1 inhibitor-based systemic therapy as first-line treatment. Patients were eligible if they had no prior anti-tumor treatment, evaluable treatment response data, and complete clinical information. Patients with incomplete clinical data, missing follow-up information, or severe comorbidities that could interfere with treatment response assessment were excluded. Peripheral blood samples were collected at baseline (C0), cycle 3 (C2), and cycle 5 (C4).

Tumor and adjacent non-tumor tissues were collected from 10 patients with hepatocellular carcinoma (HCC) at Tianjin Medical University Cancer Institute and Hospital.

All HCC patients from both the diagnostic and immunotherapy cohorts were staged according to the Barcelona Clinic Liver Cancer (BCLC) staging system (stages 0, A, B, C, and D). In addition, TNM staging was performed to assess tumor characteristics in patients without distant metastasis.

### Measurement of serum COPA

The serum COPA concentration was measured by ELISA in patients and controls. Using a commercial kit (Wuhan Meimian Biotechnology Co., Ltd, China) In summary, the COPA capture antibody was coated overnight on a 96-well ELISA microplate. After washing and incubation with blocking buffer, 10 μL of serum samples diluted fivefold were added. Following detection with the antibody and treatment with an affinity-labeled horseradish peroxidase-conjugated secondary antibody, TMB substrate solution was added to determine the amount of conjugate. Absorbance was measured at 450 nm using an enzyme-linked immunosorbent assay analyzer (MULTISKAN FC, Thermo Fisher Scientific, Shanghai). All samples were tested in duplicate.

### Measurement of serum AFP and PIVKAII

It was detected by the Roche Cobas 801 electrochemiluminescence immunoassay analyzer. The tests were performed in the Laboratory Department of the Affiliated Cancer Hospital of Tianjin Medical University.

### Immunohistochemical analysis

Formalin-fixed paraffin-embedded tissue sections were subjected to immunohistochemical staining. After deparaffinization and antigen retrieval, sections were incubated with anti-COPA antibody (Wuhan Sanying Biotechnology, Cat. No. KHC2316; dilution 1:500), followed by incubation with an HRP-conjugated secondary antibody (Zhongshan Jinqiao Biotechnology Co., Ltd., Beijing, Cat. No. KIT5020). Immunoreactivity was visualized using DAB staining, followed by hematoxylin counterstaining. COPA expression was evaluated based on staining intensity and the proportion of positive cells.

### Cohort classification and grouping criteria

For the diagnostic cohort, the optimal cutoff value for serum COPA was determined by ROC curve analysis between HCC patients and the combined control group (healthy controls and patients with chronic liver disease) using the maximum Youden index. HCC patients were subsequently classified into COPA-positive and COPA-negative groups according to this cutoff value.

For the immunotherapy cohort, treatment response was assessed according to the iRECIST (immune Response Evaluation Criteria in Solid Tumors) guidelines. Patients were classified as immune partial response (iPR), immune stable disease (iSD), or immune progressive disease (iPD) based on imaging evaluation and were further categorized into iPD and non-iPD groups for subsequent analysis of immunotherapy efficacy.

### Statistical analysis

The data of this study were analyzed using statistical software SPSS 27.0 and Prism 9.0, and other images were completed with R 4.5.0. Normality test: The Kolmogorov-Smirnov test was used (sample size >50)—non-normal distribution, i.e., two-group comparisons (Mann-Whitney U test) and multiple-group comparisons Kruskal-Wallis’s test. Agreement between radiologic and marker responses: the kappa coefficient. Using Prism 9.0 to plot the ROC curve and calculate the Youden index, the threshold corresponding to the maximum value is the optimal cut-off value. *P* < 0.05 indicated that the analysis was statistically significant.

## Results

### Characterization of COPA expression in HCC

Analysis of two independent HCC datasets (TCGA-LIHC and GSE76427) consistently demonstrated that COPA expression was significantly upregulated in HCC tissues compared with normal adjacent non-tumor tissues (Supplementary Figures S2A,B). DEGs identified from the TCGA-LIHC and GSE76427 datasets were intersected to obtain common DEGs, among which COPA showed consistent upregulation in HCC tissues. The expression patterns of the top 50 common DEGs are presented in the heatmap (Supplementary Figure S2C). Functional enrichment analysis of the common DEGs revealed significant enrichment in mitotic progression, cell cycle regulation, DNA metabolism, and translation-related biological processes (FDR < 0.05, Supplementary Figure S2D). KEGG analysis showed enrichment in cancer-related pathways, including cell cycle, DNA replication, and ribosome pathways (FDR < 0.05, Supplementary Figure S2E), which have been permitted by Kanehisa Laboratories (https://www.kegg.jp/) (Kanehisa et al., 2025; Kanehisa, 2019).

### Multi-omics expression analysis of COPA in hepatocellular carcinoma

To further validate the expression pattern of COPA, we performed multi-omics analyses using public databases. TCGA transcriptomic data demonstrated that COPA was widely expressed across multiple cancer types and showed elevated mRNA expression in HCC tissues compared with normal liver tissues. Consistently, CPTAC proteomic data revealed increased COPA protein expression in primary HCC tissues compared with normal liver tissues (Supplementary Figure S3). These findings confirmed the upregulation of COPA at both the mRNA and protein levels.

### Baseline characteristics and independent factors in the diagnostic cohort

Using the optimal cutoff value derived from ROC analysis comparing HCC patients with the combined control group, patients in the diagnostic cohort were classified into COPA-positive and COPA-negative groups. COPA positivity was significantly associated with larger tumor size (>5 cm), advanced BCLC stage (B–D), elevated AFP levels, and distant metastasis (*P* < 0.001). PIVKA-II levels also differed significantly between COPA-positive and COPA-negative groups (*P* < 0.001) (Table 1; Supplementary Figure S4).

**TABLE 1:** COPA positivity in different clinicopathological subgroups of hepatocellular carcinoma.

Variables	Total n (%)	COPA negative n (%)	COPA positive n (%)	*P* value
	N = 81	N = 17	N = 64	
Age	​	​	​	0.18
>60	43 (53.1%)	8 (18.6%)	35 (81.4%)	​
≤60	38 (46.9%)	9 (23.7%)	29 (76.3%)	​
Gender	​	​	​	0.265
Female	12 (14.8%)	4 (33.3%)	8 (66.7%)	​
Male	69 (85.2%)	13 (18.8%)	56 (81.2%)	​
Tumor size	​	​	​	<0.001
>5 cm	50 (61.7%)	6 (12%)	44 (88%)	​
≤5 cm	31 (38.3%)	11 (35.5%)	20 (64.5%)	​
BCLC stage	​	​	​	<0.001
A	27 (33.3%)	11 (40.7%)	16 (59.3%)	​
B-D	54 (66.7%)	6 (11.1%)	48 (88.9%)	​
AFP(ng/mL)	​	​	​	<0.001
>400	36 (44.4%)	2 (5.6%)	34 (94.4%)	​
≤400	45 (55.6%)	15 (33.3%)	30 (66.7%)	​
PIVKA-II(mAU/mL)	​	​	​	<0.001
>40	11 (13.6%)	5 (45.5%)	6 (54.5%)	​
≤40	70 (86.4%)	12 (17.1%)	58 (82.9%)	​
CA19-9(U/mL)	​	​	​	0.759
>50	21 (25.9%)	5 (23.8%)	16 (76.2%)	​
≤50	60 (74.1%)	12 (20%)	48 (80%)	​
FER(ng/mL)	​	​	​	0.488
>300	37 (45.7%)	6 (16.2%)	31 (83.8%)	​
≤300	44 (54.3%)	11 (25%)	33 (75%)	​
NLR	​	​	​	0.497
>3	32 (39.5%)	5 (15.6%)	27 (84.4%)	​
≤3	49 (60.5%)	12 (24.5%)	37 (75.5%)	​
AFU(U/L)	​	​	​	0.574
>50	4 (4.9%)	0 (0%)	4 (100%)	​
≤50	77 (95.1%)	17 (22.1%)	60 (77.9%)	​
HBsAg	​	​	​	0.407
Positive	68 (84%)	15 (22.1%)	53 (77.9%)	​
Negative	13 (16%)	2 (15.4%)	11 (84.6%)	​
Lymph node metastasis	​	​	​	0.229
No	73 (90.1%)	15 (20.5%)	58 (79.5%)	​
Yes	8 (9.9%)	2 (25%)	6 (75%)	​
Distant metastasis	​	​	​	<0.001
No	66 (81.5%)	17 (25.8%)	49 (74.2%)	​
Yes	15 (18.5%)	0 (0%)	15 (100%)	​
Vascular invasion	​	​	​	0.691
No	63 (77.8%)	14 (22.2%)	49 (77.8%)	​
Yes	18 (22.2%)	3 (16.7%)	15 (83.3%)	​

Data are presented as n (%). COPA positivity was defined using the optimal ROC-derived cutoff value (572.222 pg/mL). P values were calculated using the χ^2^ test or Fisher’s exact test, as appropriate. AFP, alpha-fetoprotein; PIVKA-II, protein induced by vitamin K absence or antagonist-II; CA19-9, carbohydrate antigen 19-9; FER, ferritin; NLR, neutrophil-to-lymphocyte ratio; AFU, α-L-fucosidase; HBsAg, hepatitis B surface antigen; BCLC, barcelona clinic liver cancer.

**TABLE 2:** Baseline characteristics of HCC patients receiving PD-1/PD-L1 inhibitor-based systemic therapy according to treatment response.

Variables	Non-iPD (n = 105)	iPD (n = 26)	*P* value
Age, years	52.62 ± 10.31	52.38 ± 11.40	0.919
Gender, n (%)	​	​	0.996
Male	58 (55.2)	15 (57.7)	​
Female	47 (44.8)	11 (42.3)	​
Etiology, n (%)	​	​	0.183
Alcohol-related	47 (44.8)	8 (30.8)	​
HBV infection	48 (45.7)	17 (65.4)	​
HCV infection	10 (9.5)	1 (3.8)	​
Baseline serum COPA level	433.06 ± 44.10	450.82 ± 59.41	0.034
AFP(ng/mL) median (IQR)	53.06 (5.75–1632.00)	150.45 (8.92–2346.25)	0.777
PIVKA-II(mAU/mL) median (IQR)	1472.23 (102.58–14979.00)	3667.12 (1209.84–30000.00)	0.069
BCLC stage, n (%)	​	​	0.779
B	65 (61.9)	18 (69.2)	​
C	29 (27.6)	6 (23.1)	​
D	11 (10.5)	2 (7.7)	​
Tumor size (cm) median (IQR)	6.50 (5.20–9.30)	5.65 (4.10–7.75)	0.05
Lymph node metastasis, n (%)	​	​	0.187
No	88 (83.8)	25 (96.2)	​
Yes	17 (16.2)	1 (3.8)	​
Vascular invasion, n (%)	​	​	0.298
No	98 (93.3)	22 (84.6)	​
Yes	7 (6.7)	4 (15.4)	​
AST (U/L) median (IQR)	33.00 (22.00–48.50)	27.50 (20.25–57.75)	0.853
ALT (U/L) median (IQR)	48.00 (27.50–70.00)	70.00 (33.75–94.00)	0.05
ALB (g/L)	40.30 ± 4.52	39.35 ± 5.03	0.35
TBIL (μmol/L) median (IQR)	15.60 (11.80–26.10)	20.80 (17.18–32.20)	0.007
Treatment type, n (%)	​	​	0.004
Combination therapy	92 (87.6)	16 (61.5)	​
Monotherapy	13 (12.4)	10 (38.5)	​

Data are presented as mean ± standard deviation, median (interquartile range), or number (%), as appropriate. Continuous variables were compared using Student’s t-test or Mann–Whitney U test, and categorical variables were compared using χ^2^ test or Fisher’s exact test.

Univariate analysis identified tumor size, BCLC stage, AFP, and PIVKA-II as factors associated with COPA positivity (*P* < 0.05). Multivariate analysis showed that elevated AFP remained an independent predictor of COPA positivity (OR = 6.98, 95% CI: 1.34–63.63, *P* = 0.039) (Supplementary Table S1).

### Diagnostic performance of COPA for HCC

**FIGURE 2:**
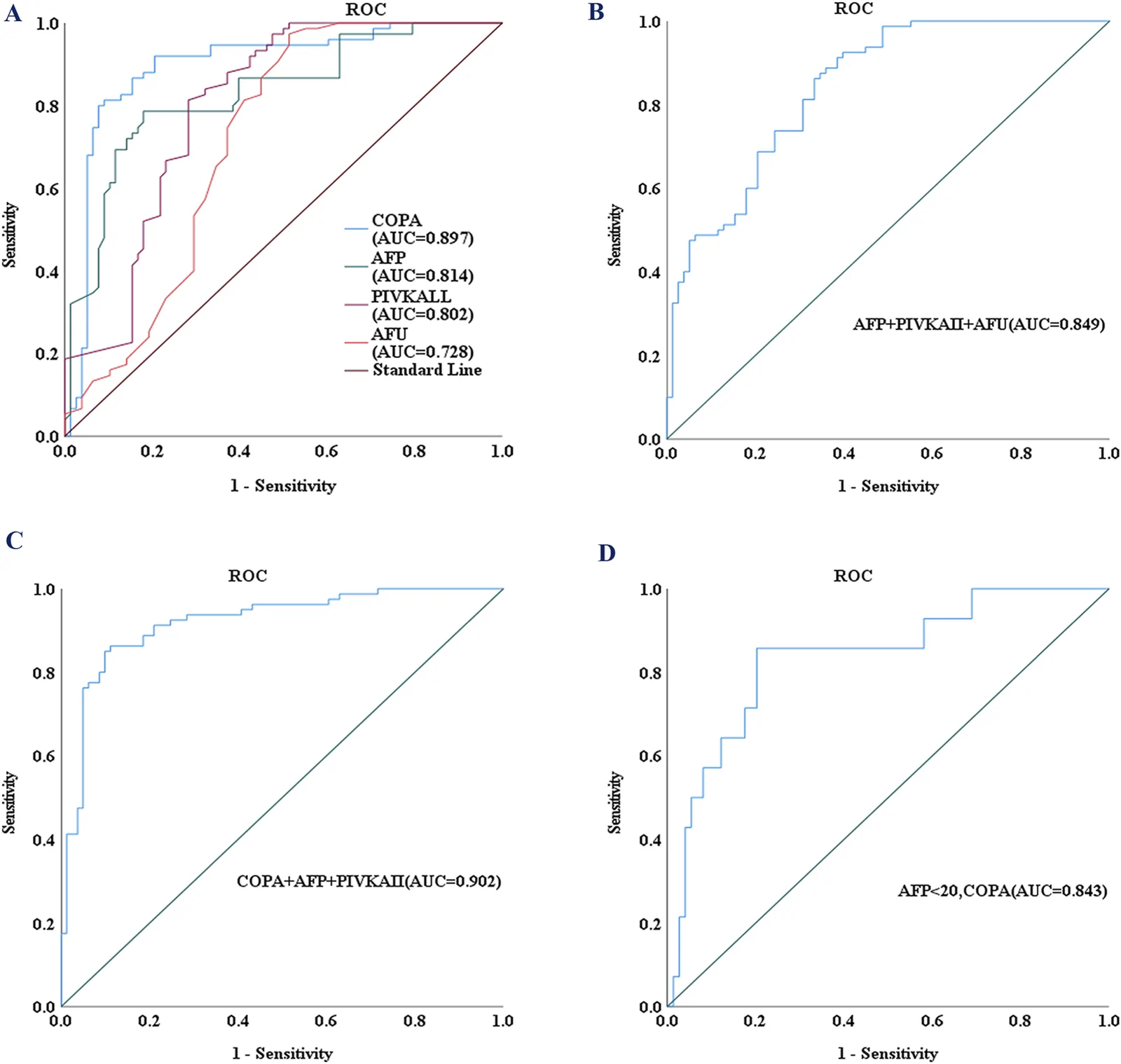
Diagnostic performance of COPA and conventional biomarkers for distinguishing HCC patients from healthy controls. **(A)** ROC curves comparing COPA with conventional biomarkers. **(B)** ROC curve of the combined model of AFU, AFP, and PIVKA-II. **(C)** ROC curve of the combined model of COPA, AFP, and PIVKA-II. **(D)** ROC curve of COPA in AFP-negative patients.

Using ROC curve analysis to distinguish HCC patients from healthy controls, COPA showed superior diagnostic performance compared with conventional biomarkers, with the highest AUC (0.897) compared with AFP (0.814), PIVKA-II (0.802), and AFU (0.728) (Figure 2A). The combination of COPA, AFP, and PIVKA-II further improved diagnostic performance (AUC = 0.902), exceeding the AFP/PIVKA-II/AFU model (AUC = 0.849) (Figures 2B,C). The AUC for COPA was 0.843 (95% CI: 0.778–0.913) in the AFP-negative (<20 ng/ml) cohort (Figure 2D).

To further assess the clinical applicability of COPA, benign liver disease patients were subsequently incorporated as additional controls. ROC analysis was repeated using HCC patients versus the combined control group (healthy controls and patients with benign liver diseases). COPA maintained strong diagnostic performance, with an AUC of 0.911 (95% CI: 0.869–0.954), which was higher than AFP (AUC = 0.826) and PIVKA-II (AUC = 0.780). Pairwise comparisons using the DeLong test demonstrated that COPA significantly outperformed AFP and PIVKA-II (Supplementary Tables S2, S3).

### COPA and immunotherapy response monitoring

A total of 131 patients with HCC receiving PD-1/PD-L1 inhibitor-based therapy were included in the immunotherapy cohort. Treatment response was evaluated according to the iRECIST criteria, and patients were classified into non-iPD and iPD groups. Baseline characteristics of the immunotherapy cohort stratified according to iRECIST response are summarized in Table 2. Most baseline clinical characteristics were comparable between the non-iPD and iPD groups. Differences were observed in baseline serum COPA levels and treatment type between the two groups.

### Dynamic changes in marker levels pre- and post-immunotherapy

**FIGURE 3:**
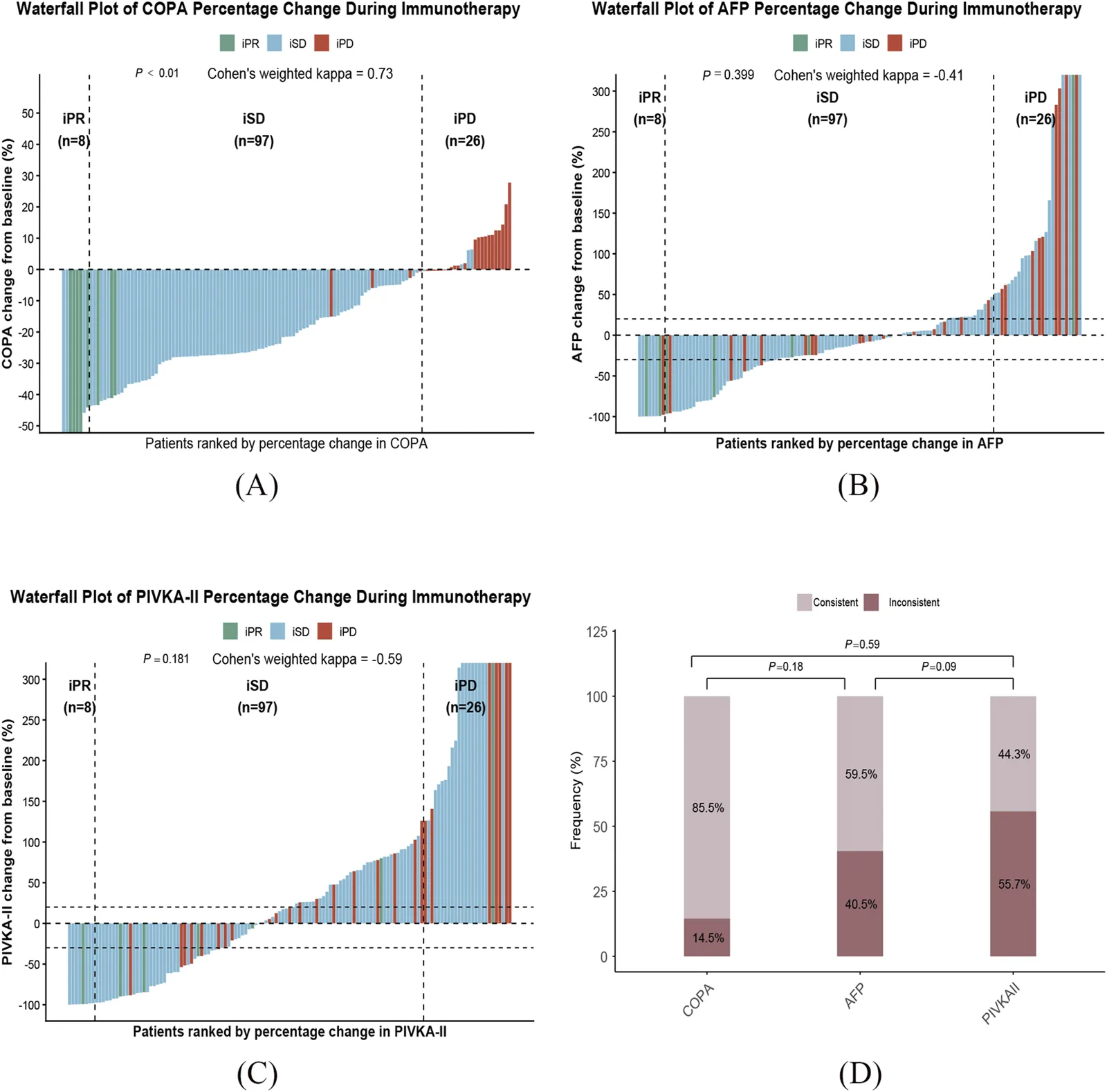
Changes in serum biomarker levels before and after immunotherapy and their concordance with treatment response. **(A–C)** The percentage changes in serum COPA, AFP, and PIVKA-II levels before and after immunotherapy were evaluated. Baseline values were defined as serum biomarker levels measured within 7 days prior to immunotherapy initiation. The concordance between biomarker changes and treatment response assessed according to the iRECIST criteria was evaluated using Cohen’s weighted kappa coefficient (Fleiss–Cohen coefficient). **(D)** Comparison of the concordance rates between changes in serum COPA, AFP, and PIVKA-II levels and immunotherapy response in 131 patients.

Changes in the levels of the three serum biomarkers were evaluated in 131 patients before and after immunotherapy. According to the iRECIST criteria, patients were classified into iSD, iPR, and iPD groups. The concordance between biomarker changes and treatment response was assessed using Cohen’s weighted kappa analysis. COPA showed a kappa coefficient of 0.73 (Figure 3A), indicating the highest consistency with treatment response compared with AFP (Figure 3B) and PIVKA-II (Figure 3C).

Subsequently, we compared the concordance rates of the three markers simultaneously elevated or diminished with imaging before and after immunotherapy (Figure 3D). COPA had the highest pre- and post-immunotherapy and imaging concordance of 85.5% compared to AFP and PIVKAII. To integrate MRI imaging, the percentage changes in three serum markers and the maximum tumor diameter before and after immunotherapy were concurrently illustrated in distinct colors for 131 patients (Supplementary Figure S5), further evidencing the correlation between COPA alterations and tumor size.

### Serum markers comparison with imaging in four cases

**FIGURE 4:**
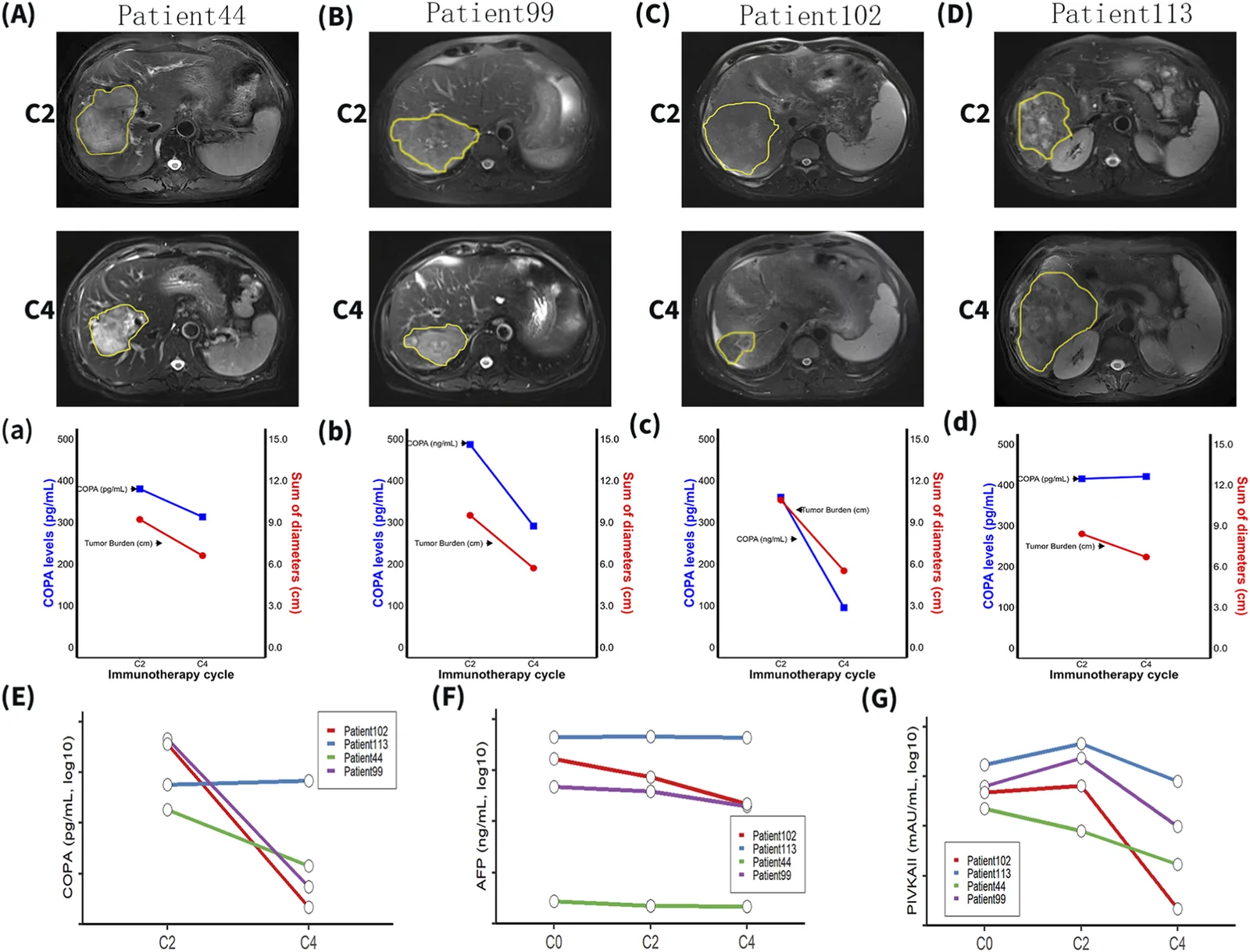
Dynamic changes of markers in HCC patients treated with immunotherapy regimens. **(A–D)** Representative MRI images of four cases at time points C2 and C4 Tumour lesion sizes before and after treatment are indicated by yellow outlines. (a–d), Changes in COPA and tumour maximum diameter before and after treatment for each of the four cases corresponding to **(A–D)**. Dynamic changes in COPA **(E)**, AFP **(F)**, and PIVKAII **(G)** before and after treatment for the four cases.

In subsequent trials, we further validated the correlation between changes in three serum biomarkers, COPA, AFP, and PIVKAII, pre- and post-immunotherapy and the tumor size as measured by imaging. Four patients (44, 99, 102, and 113) were randomly chosen and evaluated for efficacy based on the iRECIST. The target lesions in patients 44 and 99 were assessed as iSD, those in patient 102 as iPR, and those in patient 113 as iPD following stage C4. Figures 4A–D illustrates MRI imaging that depicts tumor size alterations before and after immunotherapy in four patients. A correlation exists between COPA levels and MRI findings pre- and post-immunotherapy. Specifically, there was a concurrent reduction in COPA levels in iSD and iPR patients corresponding with the regression of tumor lesions (Figures 4A-C). In contrast, COPA levels in iPD patients exhibited minimal variation alongside the progression of tumor lesions (Figure 4D). Three serum markers were compared pre- and post-treatment in each of the four patients. It was observed that patients with stable disease (iSD) and partial response (iPR) exhibited a reduction in all three markers, with a more significant decline in COPA (Figure 4E). Conversely, patients with progressive disease (iPD) showed minimal variation before and after treatment (Figures 4E–G).

### Changes in levels of long-term efficacy of markers

Subsequently, we assessed the long-term efficacy of immunotherapy in these four patients and noted disease progression. The alteration in the long-term efficacy of COPA among the four patients aligned with the trend in tumor size, whereas AFP and PIVKAII exhibited negatively correlated changes in the evaluation of long-term efficacy, indicating that serum marker levels showed less consistent trends with tumor size changes (Supplementary Figures S6A–L).

### Assessment of the level of efficacy of COPA after immunotherapy

**FIGURE 5:**
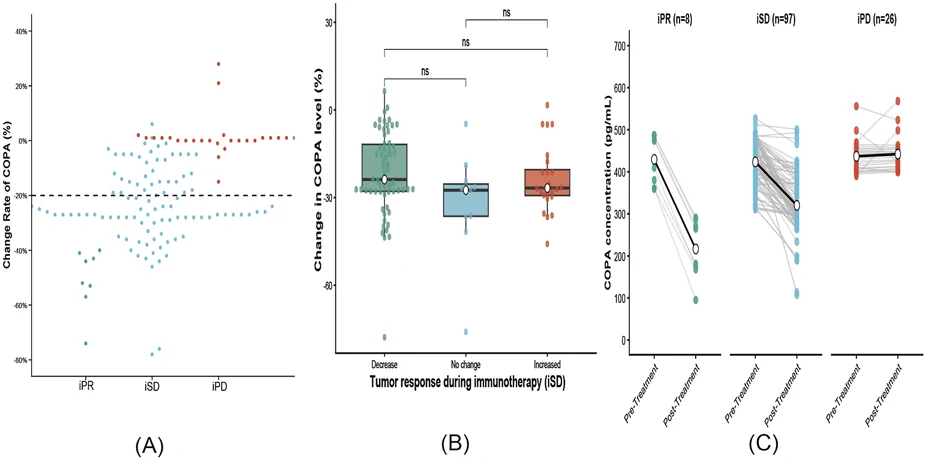
Performances of COPA in immunotherapy efficacy monitoring of HCC. **(A)** The percentage change in serum COPA levels was calculated as the difference between post-treatment and baseline COPA levels divided by the baseline value. ΔCOPA was compared among patients with iPR (n = 8), iSD (n = 97), and iPD (n = 26) according to the iRECIST criteria. **(B)** Association between changes in tumour size and ΔCOPA levels in patients with iSD after immunotherapy. **(C)** Paired line plots showing individual changes in serum COPA levels from baseline to post-treatment among patients with iPR (n = 8), iSD (n = 97), and iPD (n = 26).

In the iRECIST response evaluation criteria, when assessing treatment efficacy based on serum COPA levels, a reduction of 20% below baseline serves as a reference threshold for patients achieving partial response (iPR). Changes in COPA serum concentrations in the PR group were consistent with the efficacy assessment criteria (Figure 5A). Patients with iSD were classified according to the iRECIST criteria, which categorize tumor size into three classifications: no change post-treatment, enlargement, and reduction (Figure 5B). The trend of COPA concentration was seen to be mainly in a trend of decreasing in parallel with the tumor size. Finally, it was again verified that the change in the concentration of serum COPA before and after treatment was statistically significant in patients with SD and PR, *P* < 0.05 (Figure 5C).

### Predictive value of ΔCOPA according to treatment regimens

Patients were classified according to the iRECIST criteria. Treatment regimen was included as a covariate in both univariate and multivariate logistic regression analyses. Combination therapy was independently associated with a lower probability of iPD compared with monotherapy (OR = 0.25, 95% CI: 0.09–0.66, *P* = 0.005). The association between COPA-related variables and immunotherapy response remained significant after adjustment for treatment regimen (Supplementary Table S4).

Patients were stratified into monotherapy and combination therapy groups according to treatment regimen. Stratified ROC analysis showed that ΔCOPA exhibited predictive performance for iPD in both treatment groups, with an AUC of 0.791 (95% CI: 0.628–0.899) in the combination therapy group. ΔCOPA levels differed significantly between non-iPD and iPD patients receiving combination therapy (*P* < 0.01), whereas no significant difference was observed in the monotherapy group. In the overall cohort, ΔCOPA was also significantly different between patients with DCR and those with iPD (*P* < 0.01) (Supplementary Figure S7).

### IHC staining of COPA in hepatocellular carcinoma tissues

**FIGURE 6:**
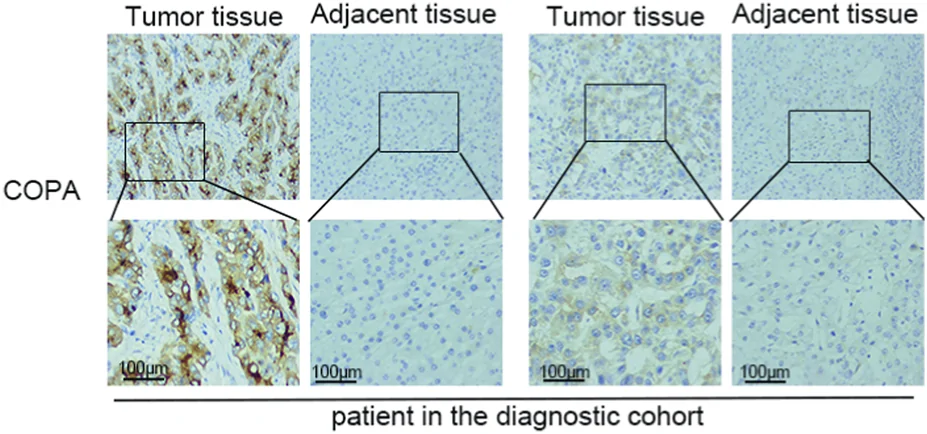
Representative immunohistochemical staining images of COPA expression in hepatocellular carcinoma tissues and adjacent non-tumor liver tissues.

Immunohistochemical staining was performed to evaluate COPA protein expression in HCC tissues and adjacent non-tumor liver tissues. COPA staining was stronger in HCC tissues than in adjacent non-tumor tissues, indicating increased COPA protein expression in HCC (Figure 6).

## Discussion

Differential expression analysis demonstrated that COPA was significantly upregulated in HCC tissues. Recent pan-cancer studies have shown that coatomer complex components are frequently dysregulated in cancer and involved in tumor progression through intracellular trafficking and oncogenic signaling regulation. As a core coatomer component, COPA may contribute to HCC development through related biological processes, including vesicular transport and cellular stress regulation (Jiang et al., 2024). Consistent with these findings, enrichment analysis of common DEGs identified from TCGA-LIHC and GSE76427 revealed significant involvement of cell cycle regulation, DNA metabolism, translation, and cancer-related pathways (FDR <0.05), suggesting that COPA dysregulation may be associated with tumor proliferation and intracellular regulatory processes in HCC. Furthermore, our GO cellular component analysis showed that COPA was enriched in cytoplasmic and Golgi-associated compartments, which is consistent with the biological function of coatomer-mediated vesicular trafficking.

Mechanistically, previous studies have demonstrated that COPA is regulated by ADAR2-mediated RNA editing, and reduced COPA editing may promote hepatocarcinogenesis by disrupting the balance between wild-type COPA and the edited COPA (I164V) variant, with downstream effects on oncogenic signaling pathways (Song et al., 2021; Chen et al., 2024). In addition, RNA editing alterations have been associated with immune cell infiltration and immunotherapy responses in HCC, providing a potential link between COPA dysregulation and tumor immune regulation (Hou et al., 2023; Bao et al., 2022; Qi et al., 2014; Chan et al., 2014). Interestingly, a recent single-cell transcriptomic study in HER2-positive gastric cancer identified COPA as a potential mediator of B lymphocyte–M2 macrophage communication, suggesting a possible role of COPA in tumor immune regulation beyond its canonical function in intracellular trafficking. Although this finding was reported in gastric cancer, it provides additional support for exploring the relationship between COPA and the immune microenvironment in HCC (Sen et al., 2026). Collectively, these findings provide a mechanistic rationale for investigating COPA as a biomarker for HCC diagnosis and immunotherapy monitoring.

Our study showed that COPA exhibited improved diagnostic performance compared with conventional biomarkers and retained high sensitivity in AFP-negative patients. Furthermore, incorporating COPA enhanced the diagnostic performance of conventional biomarker combinations, supporting its potential role as a complementary biomarker for HCC diagnosis. Compared with the multi-marker strategy proposed by Kim et al. (Kim et al., 2023) (AFP + PIVKA-II), the addition of COPA further improved diagnostic performance, suggesting that COPA may provide additional value to current HCC diagnostic approaches.

This efficacy cohort included 131 patients with HCC receiving PD-1/PD-L1 inhibitor-based systemic therapy. Combination strategies may enhance antitumor immunity by modulating the tumor microenvironment. Longitudinal analysis showed that dynamic changes in serum COPA levels were associated with immunotherapy response. Compared with conventional markers such as AFP and PIVKA-II, ΔCOPA demonstrated a stronger association with treatment outcomes. Patients achieving tumor response or disease control showed a decreasing trend in COPA levels that paralleled tumor regression, whereas patients with progressive disease exhibited distinct dynamic patterns. Furthermore, subgroup analysis according to treatment regimen demonstrated better predictive performance of ΔCOPA in patients receiving combination therapy than in those receiving monotherapy, suggesting its potential value for immunotherapy response monitoring across different treatment settings.

Therefore, this study applies serum COPA to the diagnosis of hepatocellular carcinoma and the monitoring of immunotherapy efficacy. Not only can it achieve a definitive diagnosis of hepatocellular carcinoma, but it also enables comprehensive analysis by integrating COPA expression levels with the patient’s clinical staging. Serum COPA levels also showed dynamic changes before and after immunotherapy, correlating with alterations in tumor volume. This strategy demonstrates its potential for clinical application and translation, and provides an important foundation for subsequent related research.

This study has limitations: the limited sample size during the research period and patient follow-up loss hindered a comprehensive assessment of COPA’s long-term predictive capability and failed to clarify its specificity for hepatocellular carcinoma subtypes (Expert Committee, 2024). The extended research cohort is currently being updated, but the timing of the update remains uncertain. More comprehensive long-term medical records are required to fully evaluate predictive value of COPA over time. Follow-up studies are planned to address specific areas requiring improvement. Future studies will focus on comprehensively addressing these limitations. Additionally, heterogeneity among different PD-1/PD-L1 inhibitors (e.g., sintilimab and durvalumab) and combination regimens (e.g., with bevacizumab) may impact biomarker stability. Variations in COPA expression across different populations may also influence study outcomes. Additionally, heterogeneity among different PD-1/PD-L1 inhibitor-based treatment regimens may influence biomarker performance. Although subgroup analyses were performed, the retrospective design and limited sample size require further validation in larger prospective studies.

Future cohort extension and prolonged follow-up are necessary to validate the functional mechanism of COPA in conjunction with organoid modelling (Chan et al., 2024). The interaction of COPA with other cytosolic subunits was thoroughly examined using single-cell sequencing and spatial transcriptome technologies (Wong et al., 2003; Greten et al., 2023). Additionally, certain studies have indicated that targeting immunosuppressive cells in the tumor microenvironment markedly improves the efficacy of PD-1 inhibitors (Tu et al., 2024). Future investigations into the interactions between COPA and this cell population may facilitate the development of combination therapy strategies. Moreover, the newly suggested method of extracellular vesicle enrichment offers a novel strategy for liquid biopsy applications of COPA proteins (Zhang et al., 2022), potentially addressing the shortcomings of tissue samples.

## Conclusion

This study demonstrates that COPA exhibits superior diagnostic performance compared with AFP and PIVKA-II and maintains excellent diagnostic accuracy even in AFP- and PIVKA-II-negative patients. Dynamic changes in COPA during immunotherapy are in good agreement with imaging-based assessment of solid tumor response, outperforming conventional biomarkers. Collectively, these findings suggest that COPA may provide an integrated approach for both the diagnosis and treatment monitoring of hepatocellular carcinoma.

## Data Availability

The original contributions presented in the study are included in the article/Supplementary Material, further inquiries can be directed to the corresponding authors.
