# Supplementary material for: Serum COPA for the diagnosis and immunotherapeutic monitoring of hepatocellular carcinoma: a retrospective study
Source: Front Pharmacol. 2026 Aug 21;17:1908605. doi: 10.3389/fphar.2026.1908605 (PMC13543294; doi:10.3389/fphar.2026.1908605)
Supplement: Supplementary file 1 [file DataSheet1.pdf]

Serum COPA for the diagnosis and immunotherapeutic monitoring of hepatocellular carcinoma: a retrospective study

Ranliang Cui [1#],Yue Cao[2#] ,Li Ren [1*], Yueguo Li [1*]

Supplementary Materials & Methods

ELISA

Serum COPA assay: double antibody sandwich ELISA; kit information: human COPA protein ELISA scientific research kit ELISA scientific research kit; Item No.: MM-64849H1/MM-50499H1. The basic operation procedure is as follows: prepare reagents, samples and standards -add the prepared samples and standards, and react for 30 minutes at 37°C. -Wash the plate 5 times, add the enzyme labeling reagent and react at 37°C for 30 minutes -Add the termination solution -Read the OD value within 15 minutes -Calculate.

ABBOTT i2000

HbsAg was detected by ABBOTT i2000 chemiluminescence immunoassay analyzer. The tests were performed at the Laboratory of the Affiliated Cancer Hospital of Tianjin Medical University.

Table S1. Logistic regression analysis of factors associated with COPA positivity in patients with hepatocellular carcinoma

Variables	Univariate analysis OR (95% CI)	*P* value	Multivariate analysis OR (95% CI)	*P* value
Age (>60 vs ≤60)	1.36 (0.46–4.05)	0.576		
Gender (Male vs Female)	2.15 (0.51–8.02)	0.263		
Tumor size (>5 cm vs ≤5 cm)	4.03 (1.34–13.19)	**0.015**	2.43 (0.66–9.38)	0.182
BCLC stage (B–D vs A)	5.50 (1.80–18.32)	**0.004**	3.51 (0.98–13.32)	0.056
AFP (>400 ng/mL vs ≤400 ng/mL)	8.50 (2.16–56.78)	**0.007**	6.98 (1.34–63.63)	**0.039**
PIVKA-II (>40 mAU/mL vs ≤40 mAU/mL)	0.25 (0.06–0.98)	**0.042**	0.17 (0.02–1.03)	0.065
CA19-9 (>50 U/mL vs ≤50 U/mL)	0.80 (0.25–2.83)	0.712		
Ferritin (>300 ng/mL vs ≤300 ng/mL)	1.72 (0.58–5.52)	0.337		
NLR (>3 vs ≤3)	1.75 (0.58–6.04)	0.342		
HBsAg (Positive vs Negative)	0.64 (0.09–2.74)	0.591		
Lymph node metastasis (Yes vs No)	0.78 (0.16–5.66)	0.77		
Vascular invasion (Yes vs No)	1.43 (0.40–6.79)	0.611		

Note: OR, odds ratio; CI, confidence interval; AFP, alpha-fetoprotein; PIVKA-II, protein induced by vitamin K absence or antagonist-II; BCLC, Barcelona Clinic Liver Cancer; NLR, neutrophil-to-lymphocyte ratio; HBsAg, hepatitis B surface antigen. Univariate and multivariate logistic regression analyses were performed to identify factors associated with COPA positivity. Variables with P <0.10 in univariate analyses or with clinical relevance were included in the multivariate logistic regression model. Results are presented as odds ratios (ORs) with 95% confidence intervals (CIs).

Table S2.Diagnostic performance of AFP, PIVKA-II, and COPA for distinguishing HCC from the combined control group.

Biomarker	AUC (95% CI)	Optimal cutoff	Sensitivity	Specificity
AFP	0.826 (0.766–0.886)	8.38	70.40%	84.70%
PIVKA-II	0.780 (0.707–0.854)	145.77	64.20%	94.10%
COPA	0.911 (0.869–0.954)	422.222	95.10%	83.90%

Note: Combined control group (healthy controls and patients with chronic liver disease)

Table S3.DeLong test comparing AUCs of biomarkers

Comparison	Z value	*P* value
COPA vs AFP	2.137	0.0326
COPA vs PIVKA-II	2.931	0.00337
AFP vs PIVKA-II	1.23	0.219
AFP-negative: COPA vs PIVKA-II	4.904	<0.001
PIVKA-II-negative: COPA vs AFP	3.107	0.00189

Table S4. Univariate and multivariate logistic regression analyses of factors associated with progressive disease after immunotherapy

Variables	Univariate analysis OR (95% CI)	*P* value	Multivariate analysis OR (95% CI)	*P* value
COPA baseline level (continuous)	0.99 (0.98–1.00)	**0.038**	0.99 (0.98-1)	**0.026**
Age (years)	1.00 (0.96–1.04)	0.822		
Gender (Male vs Female)	0.93 (0.39–2.15)	0.865		
Etiology	2.08 (0.84-5.53)	0.123		
HBV infection vs Alcohol-related	2.43 (0.99–6.39)	0.059		
HCV infection vs Alcohol-related	0.59 (0.03–3.76)	0.634		
AFP group (>400 vs ≤400 ng/mL)	0.87 (0.37–2.09)	0.743		
PIVKA-II group (>40 vs ≤40 mAU/mL)	0.34 (0.05–1.28)	0.165		
BCLC stage (C–D vs B)	0.78 (0.31–1.85)	0.578		

**Tumor size (>5 cm vs ≤5 cm)**	1.06 (0.44–2.48)	0.891		
Lymph node metastasis (Yes vs No)	0.19 (0.01–0.98)	0.112		
Vascular invasion (Yes vs No)	2.29 (0.56–8.23)	0.215		
AST (U/L)	1.01 (1.00–1.02)	0.137		
ALT (U/L)	1.01 (1.00–1.01)	0.055		
ALB (g/L)	0.94 (0.85–1.03)	0.16		
TBIL (μmol/L)	1.01 (1.00–1.02)	0.1		
Treatment type				
Combination therapy vs Monotherapy	0.26 (0.10–0.69)	**0.006**	0.25 (0.09–0.66)	**0.005**

Note: ORs were calculated using logistic regression analysis. Combination therapy was compared with monotherapy as the reference group. COPA baseline level was analyzed as a continuous variable. Variables with clinical relevance were included in multivariate analysis. OR, odds ratio; CI, confidence interval; COPA, coatomer subunit alpha; HBV, hepatitis B virus; HCV, hepatitis C virus; AFP, alpha-fetoprotein; PIVKA-II, protein induced by vitamin K absence or antagonist-II; BCLC, Barcelona Clinic Liver Cancer; AST, aspartate aminotransferase; ALT, alanine aminotransferase; ALB, albumin; TBIL, total bilirubin.

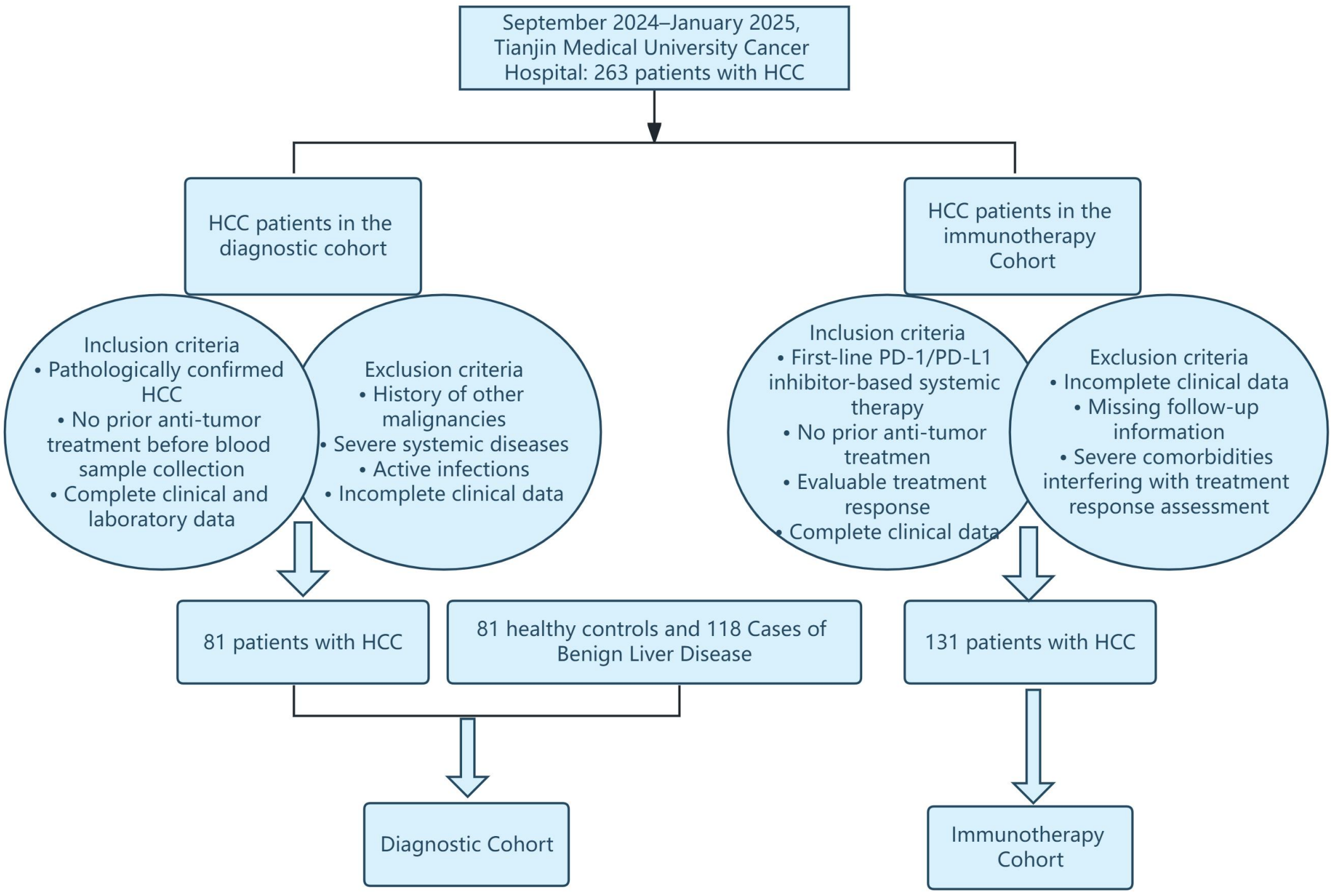

Supplementary Fig. 1 Flowchart of included

participants.

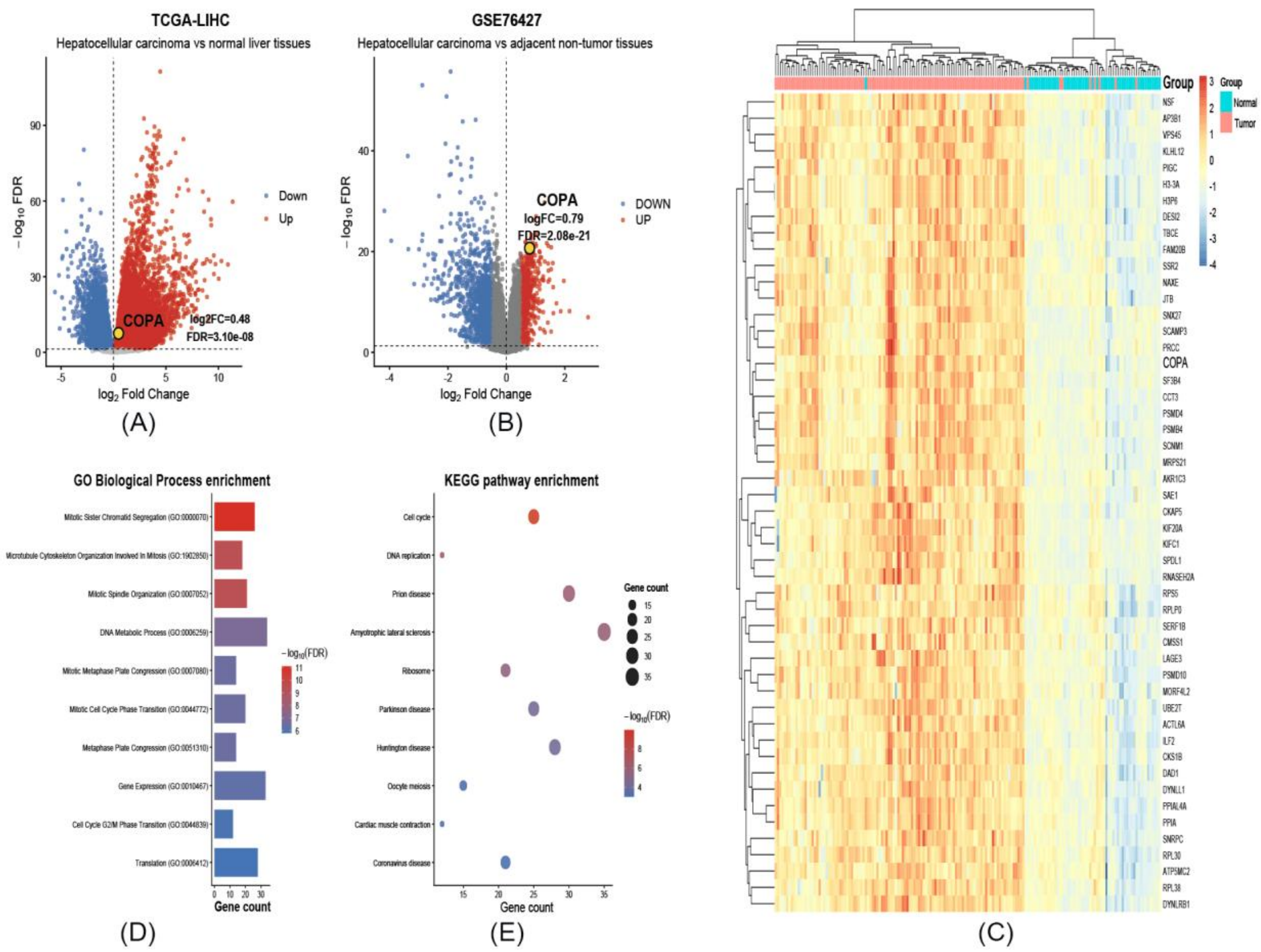

Supplementary Fig. 2 Expression profiling of COPA and common differentially expressed genes (DEGs) identified from two independent HCC datasets.

(A) Volcano plot showing DEGs between hepatocellular carcinoma and normal liver tissues in the TCGA-LIHC cohort. Red dots indicate upregulated genes, and blue dots indicate downregulated genes. (B) Volcano plot showing DEGs between HCC and paired adjacent non-tumour tissues in the GSE76427 dataset. (C) Heatmap displaying the top 50 common DEGs identified by intersecting DEGs from the TCGA-LIHC and GSE76427 datasets. (D) Bar plot showing GO Biological Process enrichment analysis of the common DEGs from the TCGA-LIHC and GSE76427 datasets. The x-axis indicates the number of enriched genes. (E) Bubble plot showing KEGG pathway enrichment analysis of the common DEGs. Bubble size indicates gene count. (https://www.kegg.jp/).

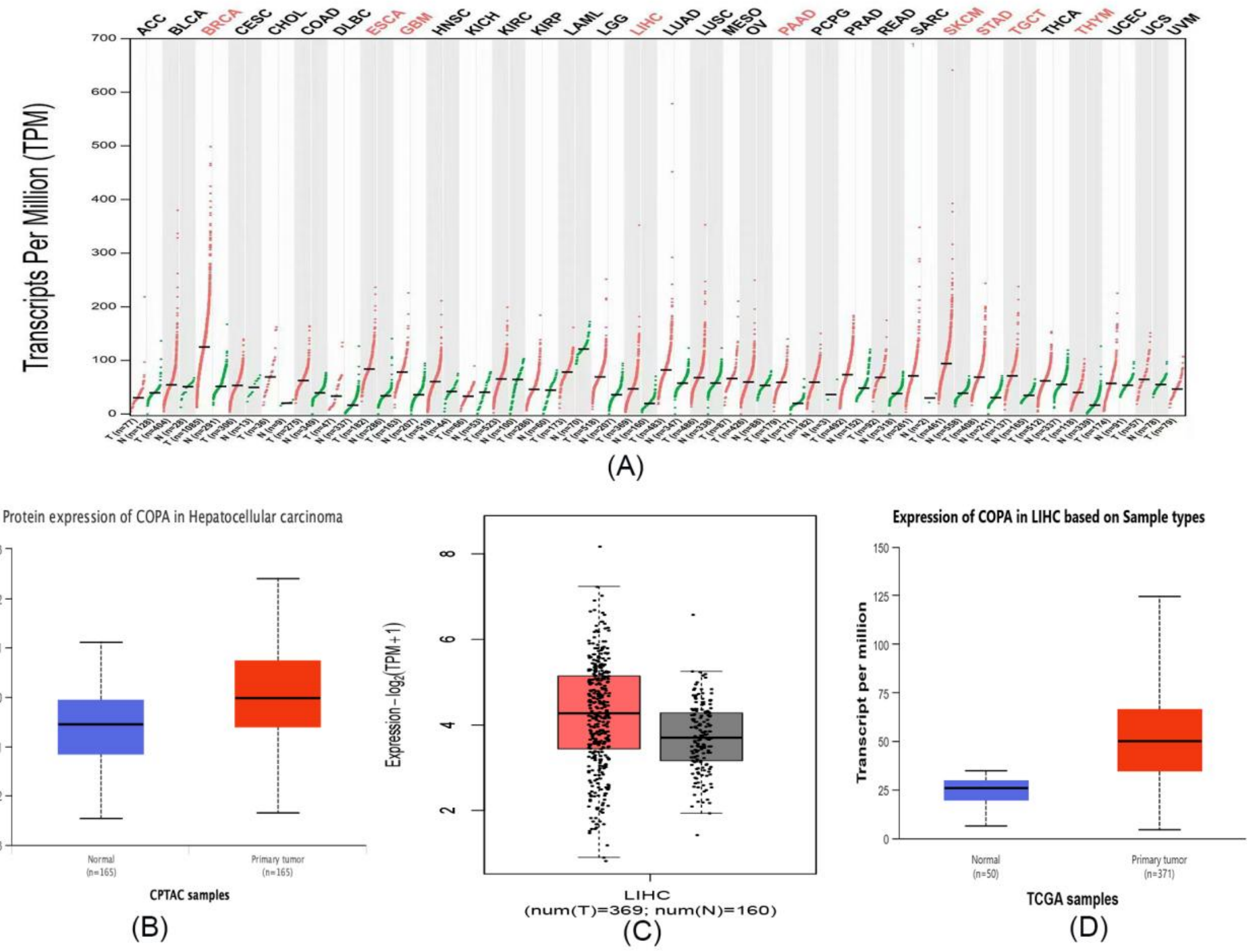

Supplementary Fig. 3 Multi-omics expression analysis of COPA in pan-cancer and hepatocellular carcinoma. (A) Pan-cancer mRNA expression profile of COPA based on TCGA transcriptomic data. The y-axis represents gene expression levels quantified as TPM. (B) COP A protein expression analysis in normal liver and primary HCC tissues based on CPTAC proteomic data. Protein expression levels are presented as Z-scores. (C) Comparison of COP An mRNA expression between primary HCC tissues and normal liver tissues in the TCGA-LIHC cohort from the TCGA database (369 tumour samples and 160 normal samples). (D) Comparison of COPA transcript levels between normal liver tissues and primary HCC tissues based on TCGA transcriptomic data (TPM values).

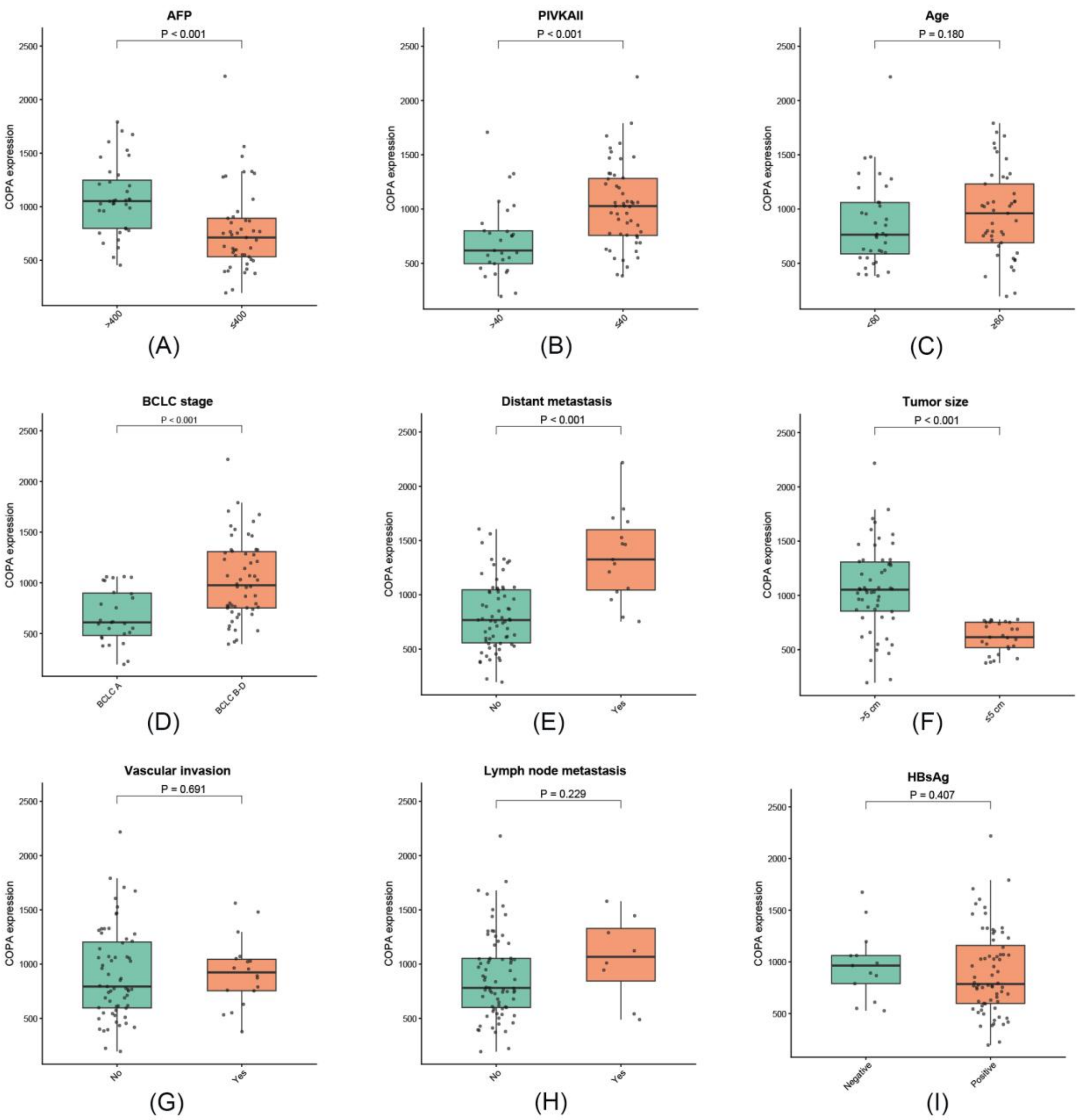

Supplementary Fig. 4 COPA expression levels across diverse clinical subgroups in hepatocellular carcinoma (HCC). (A–I) COPA positivity rates were compared according to AFP level, PIVKA-II level, age, BCLC stage, distant metastasis, tumour size, vascular invasion, lymph node metastasis, and HBsAg status, respectively. P values are indicated in the corresponding panels.

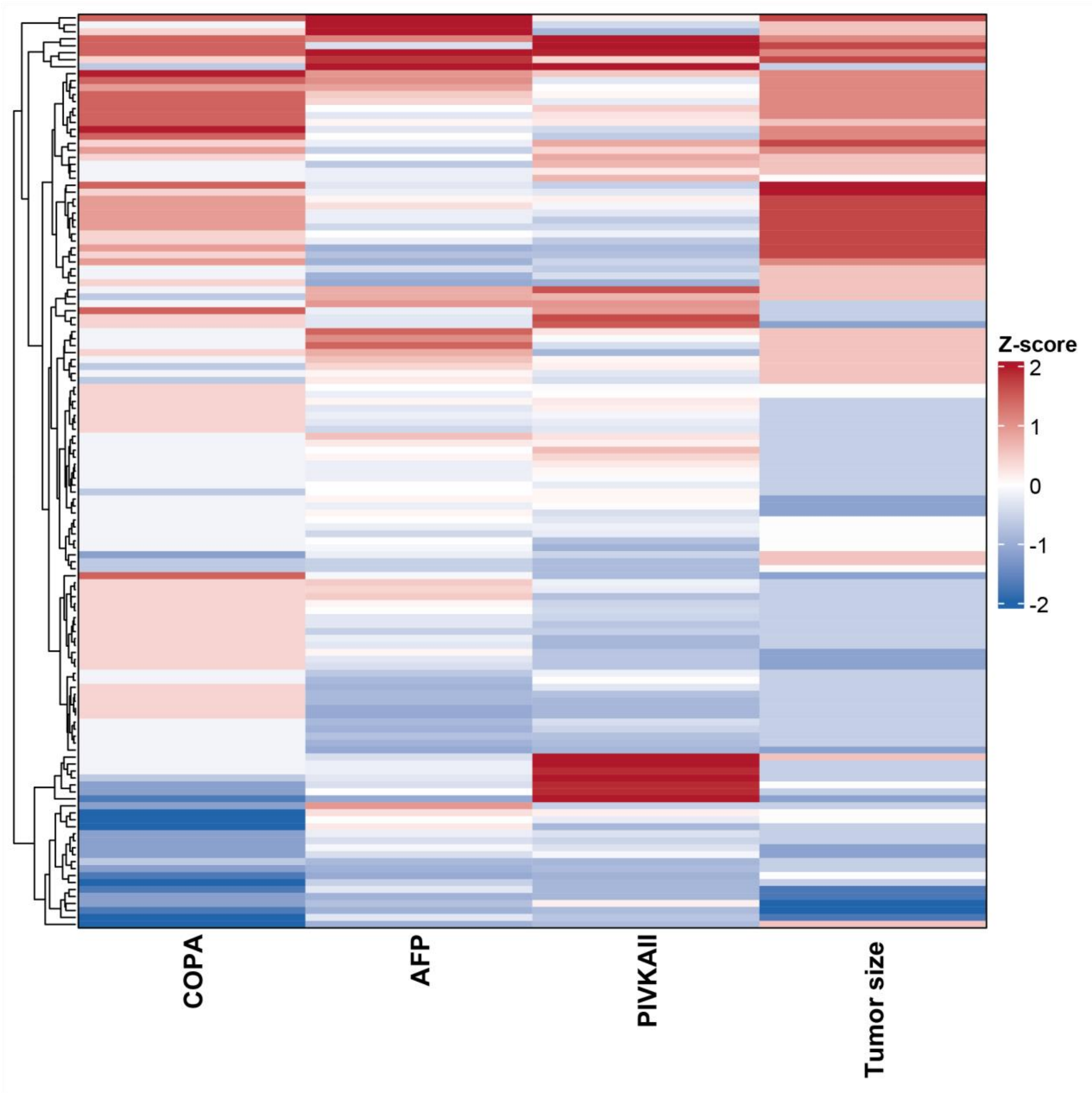

Supplementary Fig. 5 Changes in serum biomarker levels and tumor size before and after immunotherapy in 131 patients with HCC. Heatmap showing the changes in serum COPA, AFP, PIVKA-II levels, and tumor size before and after immunotherapy. Each row represents an individual HCC patient, and columns represent serum COPA, AFP, PIVKA-II, and tumor size. The values were normalized using Z-score transformation, with blue, white, and red indicating relatively low, intermediate, and high levels, respectively.

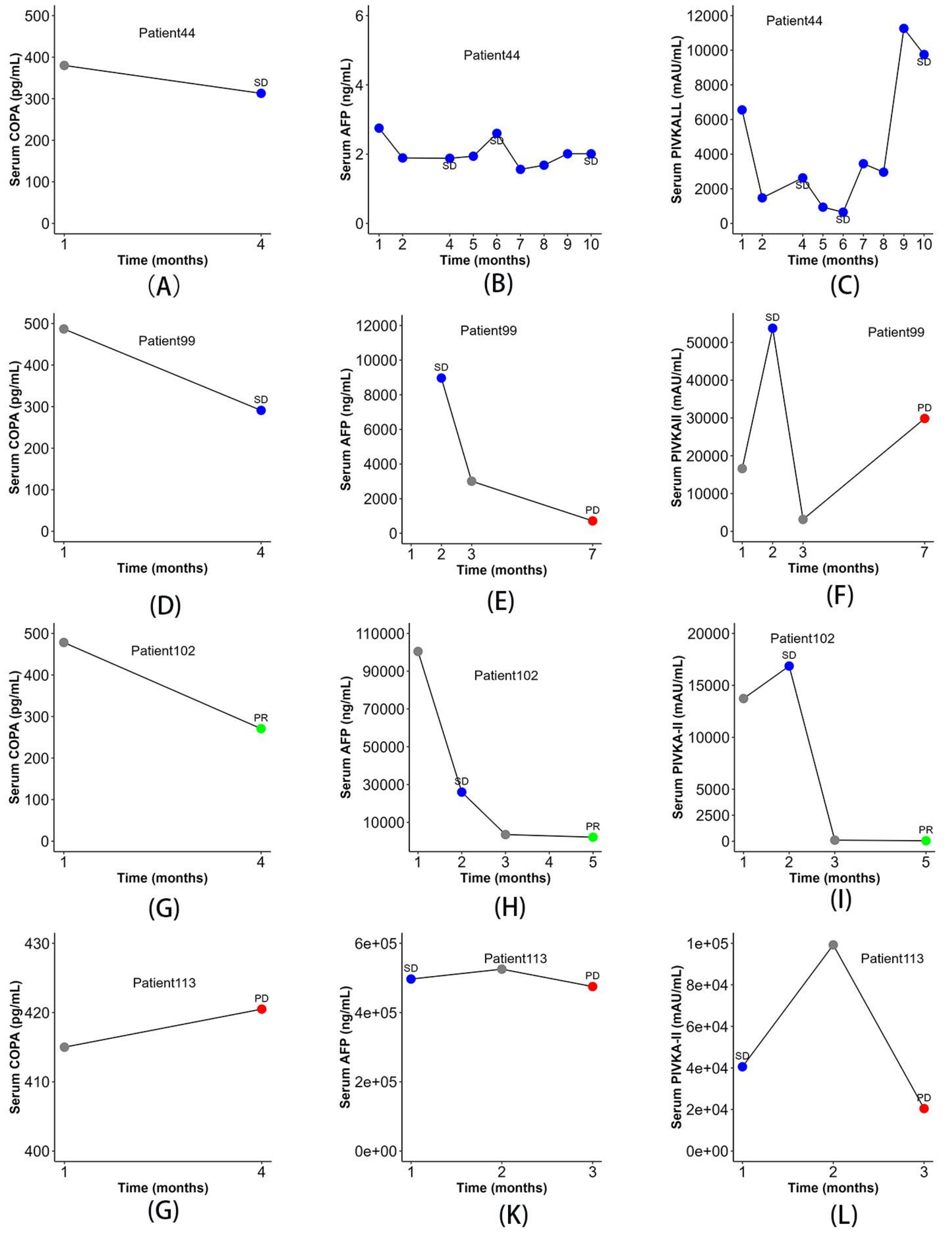

Supplementary Fig.6 Performance s in the markers of long-term immunotherapy efficacy monitoring of HCC patients.(A-C) COPA, AFP, and PIVKAII of Patient44. (**D-F)** COPA, AFP, and PIVKAII of Patient99. **(G-I**）COPA, AFP, and PIVKAII of Patient102. (**J-L)** COPA, AFP, and PIVKAII of Patient113.

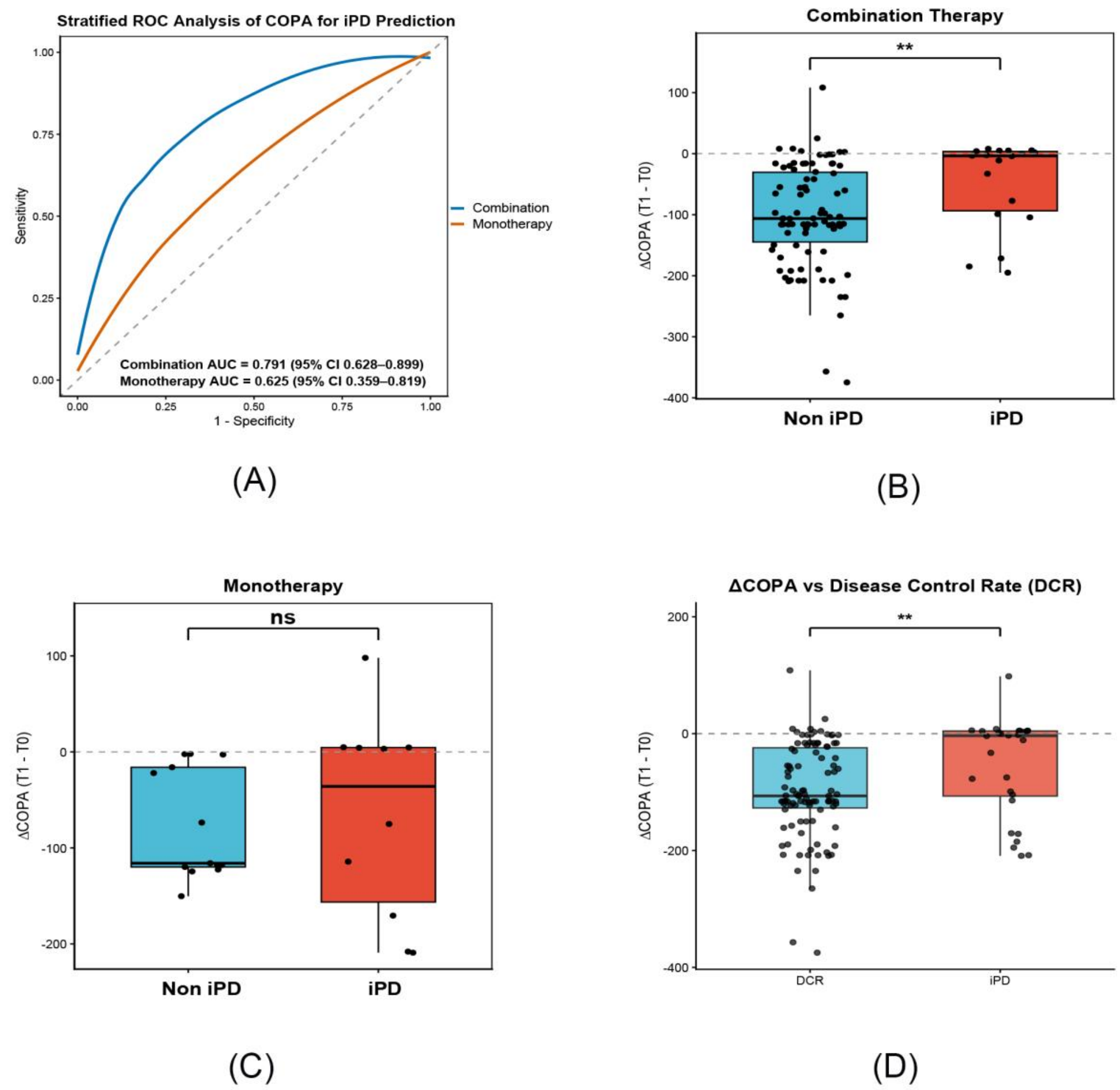

Supplementary Fig.7 Predictive value of dynamic COPA changes during PD-1/PD-L1 inhibitor-based therapy. (A) Stratified ROC curve analysis showing the predictive performance of ΔCOPA for identifying iPD patients in the combination therapy and monotherapy groups. **(B)** Comparison of ΔCOPA between non-iPD and iPD patients receiving combination therapy. **(C)** Comparison of ΔCOPA between non-iPD and iPD patients receiving monotherapy. **(D)** Comparison of ΔCOPA between patients with disease control and those with iPD in the overall immunotherapy cohort. Note: ΔCOPA was calculated as the difference between serum COPA levels after treatment and baseline levels. **Note:** ΔCOPA was calculated as the difference between serum COPA levels after treatment and baseline levels.
